# Clinical study of children with cryofibrinogenemia: a retrospective study from a single center

**DOI:** 10.1186/s12969-018-0249-6

**Published:** 2018-04-24

**Authors:** Hsiao-Feng Chou, Yu-Hung Wu, Che-Sheng Ho, Yu-Hsuan Kao

**Affiliations:** 1 Song Zhan Clinics, No.88, Songlong Rd, Taipei City, 110 Taiwan; 2 Department of Dermatology, Mackay Memorial Hospital, No. 92, Sec. 2, Zhong-shan N. Rd, Taipei City, 10449 Taiwan; 3 Section of Neurology, Department of Pediatrics, Mackay Memorial Hospital, No. 92, Sec. 2, Zhong-shan N. Rd, Taipei City, 10449 Taiwan; 4 Section of Immunology, Rheumatology and Allergy, Department of Pediatrics, Mackay Memorial Hospital, No. 92, Sec. 2, Zhong-shan N. Rd, Taipei City, 10449 Taiwan

**Keywords:** Cryofibrinogenemia, Children, Central nervous system

## Abstract

**Background:**

This study aimed to evaluate the demographic, clinical features, laboratory data, pathology and other survey in pediatric patients with cryofibrinogenemia.

**Methods:**

A 12-year retrospective chart review identified eight pediatric patients at Mackay Memorial Hospital, Taipei, Taiwan.

**Results:**

The female-to-male ratio was 3:1. The mean age at symptom onset and of diagnosis was 10.3 ± 4.6 years and 12.3 ± 4 years, respectively. One child (12.5%) had primary cryofibrinogenemia. The common symptoms were purpura, arthralgia, and muscle weakness (100%). On laboratory examination, cryofibrinogen was positive in all patients. All patients had increased anti-thrombin III while 87.5% and 62.5% had abnormal protein S and protein C, respectively. All eight also complained of neurologic symptoms. One had vertebral artery narrowing, two showed increased T2-weighted signal intensity on the thalamus or white matter, and one had acute hemorrhagic encephalomyelitis on brain magnetic resonance imaging.

**Conclusions:**

This study reports on the presentations of cryofibrinogenemia, which is rare in children. Most cases are associated with autoimmune disease and have severe and complex presentations. Central nervous system involvement is common.

## Background

Cryofibrinogenemia is a rare hematologic disorder wherein plasma forms a cryoprecipitate that consists of fibrinogen, fibrin, fibronectin, factor VII, and smaller amounts of various plasma proteins. Cryofibrinogen precipitates at a low temperature (4 °C) and re-dissolves on warming to (37 °C) [1]. Its exact pathogenesis has not been elucidated. A first hypothesis is that there is a defect in the fibrinolytic process, with high plasma levels of α1 antitrypsin and α2-macroglobulin (protease inhibitors), and delayed lysis of euglobulin. But since elevated plasma α1-antitrypsin and α2-macroglobulin levels are not found in other studies, a second hypothesis favors an increased thrombin-binding capacity and clot formation. Both hypotheses lead to thrombotic occlusions of medium and small vessels, leading to amplified hyper-viscosity, reflex vasospasms, and vascular stasis [2].

The most common symptoms of cryofibrinogenemia are due to cutaneous ischemia and include purpura, livedo reticularis, blisters, tissue necrosis, and ulceration. Systemic symptoms have been reported to be glomerulonephritis and thrombophlebitis [3]. Cases involving children have rare been reported. This is a retrospective review on pediatric patients diagnosed with cryofibrinogenemia in a tertiary medical center in Taiwan.

## Methods

After a review of the diagnostic criteria and clinical features, a medical chart review of eight patients younger than 18 years with a diagnosis of cryofibrinogenemia at the pediatric department of Mackay Memorial Hospital, Taipei, Taiwan, between May 2010 and July 2015. The diagnostic criteria originally proposed by essential and supportive evidence. Essential evidence included: 1) appropriate clinical presentation of sudden onset of skin changes and constitutional symptoms, with or without thrombosis, bleeding, or exposure to cold; 2) presence of cryofibrinogen in plasma; and 3) absence of cryoglobulins. Essential cryofibrinogenemia meant no secondary causes of cryofibrinogens and no evidence of other vaso-occlusive diseases.

Supportive evidence was non-specific and not compulsory. However, when present along with essential evidence, supportive evidence improved diagnostic accuracy. Supportive evidence included: 1) angiogram with abrupt occlusion of small to medium sized arteries; 2) typical skin biopsy findings of cryofibrinogen plugging vessels, leukocytoclastic vasculitis, or dermal necrosis; and 3) elevated serum levels of α1-antitrypsin and α2-macroglobulin [3].

Data were collected and charted in terms of sex, age at onset, initial complaints, presenting features, laboratory values, electrophysiologic examinations, imaging studies (i.e., angiography and magnetic resonance imaging [MRI]), and skin biopsy.

The clinical symptoms evaluated were defined as follows: 1) skin manifestations, including purpura, Raynaud phenomenon, skin necrosis or ulceration, livedo reticularis, urticaria, gangrene, sensitivity to cold, and ecchymosis; 2) neurologic involvement (e.g. muscle weakness, numbness, headache, vertigo, dizziness, seizures, dysuria, and dysphonia); 3) arthralgia, fever, and myalgia; and 4) other systemic symptoms like dyspnea, chest pain, abdominal pain, hematochezia, menorrhea, bone pain, or insomnia.

The laboratory examinations included platelet count, Prothrombin time (PT), activated partial thromboplastin time (aPTT), von Willebrand factor (vWF), serum immunoglobulin A (IgA), cryofibrinogen, cryoglobulin, α1 anti-trypsin, and other immunological examinations. Hepatitis C virus (HCV) antigen, anti-HCV, hepatitis B virus (HBV) surface antigen (HBsAg), and anti-HBV antibodies were measured. Proteinuria, hematuria, and serum creatinine were also recorded.

Systemic involvements were evaluated by several examinations. Skin biopsy was performed for establish the diagnosis. Arthritis indicated joint swelling with two or more of erythema, local heat, tenderness, or limited range of motion. In the peripheral and central nervous system (CNS), neuritis indicated peripheral neuropathy on nerve conduction velocity (NCV), while patients presented with numbness, weakness, or pain. Brain and spine MRI, electromyogram (EMG), somato-sensory evoked potential (SSEP), brainstem auditory evoked potential (BAEP), and visual evoked potential (VEP) were performed according CNS symptoms and signs.

In the respiratory system, pulmonary embolism and acute respiratory distress syndrome (ARDS) were recorded. Renal involvement was indicated by proteinuria, hematuria, or elevated serum creatinine.

Arterial thrombosis was confirmed clinically and by angiogram of the lower limbs, superior mesentery artery, and celiac artery. The associated autoimmune diseases were diagnosed based on the following criteria: systemic lupus erythematosus (SLE) was defined accorded to the 1997 revised criteria of the American College of Rheumatology [4]; juvenile idiopathic arthritis (JIA) was defined by the International League of Associations for Rheumatology criteria [5]; juvenile dermatomyositis (JDM) was according to the criteria of Bohan and Peter in 1975 [6]; Behcet’s disease was diagnosed according to the International Study Group for Behcet’s disease in 1990 [7] and cutaneous mastocytosis was according to the criteria of a consensus proposal of Valent et al. in 2001 [8].

## Results

**Fig. 1:**
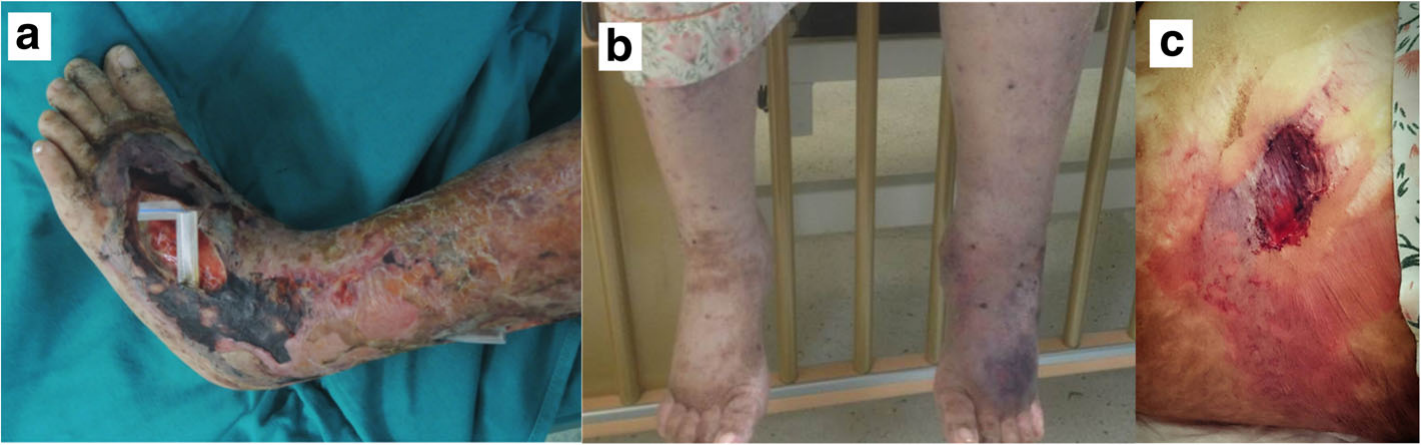
**a**) Extensive purpura and gangrene on the left foot (Case 7). **b**) Ecchymosis and swelling on the left foot (Case 3). **c**) Ulcer on the buttocks (Case 3)

The female-to-male ratio was 3:1. The mean age at symptom onset was 10.3 ± 4.6 months (range, 2.5–18.5 years). The mean age at diagnosis was 12.3 ± 4 months (range, 4–18.6 years). Based on the symptoms identified, the most common initial symptoms were arthralgia and myalgia. The most common symptoms were purpura, muscle weakness, and arthralgia (Table 1). The skin manifestations were shown in Fig. 1.

**Table 1:** Clinical symptoms and demographic features children

Case	1		2 (primary)		3		4		5		6		7		8		Total	
Sex	M		F		F		F		M		F		F		F		Female/male ratio 3:1	
Age of onset (yrs)	9.4		13.6		11.4		9.6		2.5		12.4		18.5		14.6		Mean (SD) age at on set (yrs) 10.3 ± 4.6	
Age at diagnosis (yrs)	9.6		13.8		11.6		10.6		4		13.6		18.6		17.1		Mean (SD) at diagnosis (yrs) 12.3 ± 4	
Expired on (yrs)	10.4		Loss to follow up		12.1		10.8		(−)		(−)		19		17.6			
Clinical symptoms																	All patients	
Initial/Total																	Initial, n	Total, n
Myalgia	(+)	(+)	(+)	(+)	(+)	(+)	(+)	(+)	(−)	(−)	(−)	(+)	(−)	(+)	(+)	(+)	5	7
Fever	(−)	(+)	(−)	(+)	(−)	(+)	(−)	(−)	(−)	(−)	(−)	(−)	(−)	(+)	(−)	(−)	0	4
Arthralgia	(+)	(+)	(+)	(+)	(+)	(+)	(+)	(+)	(+)	(+)	(+)	(+)	(−)	(+)	(+)	(+)	7	8
Skin manifestations																		
Purpura	(−)	(+)	(+)	(+)	(−)	(+)	(+)	(+)	(−)	(+)	(−)	(+)	(+)	(+)	(−)	(+)	3	8
Raynaud phenomenon	(−)	(+)	(−)	(−)	(−)	(−)	(−)	(−)	(−)	(−)	(−)	(−)	(−)	(−)	(−)	(−)	0	1
Skin necrosis or ulceration	(−)	(+)	(−)	(−)	(−)	(+)	(−)	(−)	(+)	(+)	(−)	(−)	(−)	(+)	(−)	(+)	1	5
Livedo reticularis	(−)	(+)	(−)	(−)	(+)	(+)	(−)	(−)	(−)	(−)	(−)	(−)	(−)	(+)	(−)	(−)	1	3
Urticaria	(−)	(−)	(+)	(+)	(−)	(−)	(−)	(−)	(−)	(−)	(−)	(−)	(−)	(+)	(+)	(+)	2	3
Gangrene	(−)	(+)	(−)	(−)	(−)	(+)	(−)	(−)	(−)	(−)	(−)	(−)	(−)	(+)	(−)	(+)	0	4
Sensitivity to cold	(−)	(−)	(+)	(+)	(−)	(−)	(−)	(+)	(−)	(−)	(−)	(+)	(−)	(+)	(−)	(+)	1	5
Ecchymosis	(−)	(+)	(−)	(−)	(−)	(+)	(−)	(+)	(−)	(+)	(−)	(+)	(+)	(+)	(−)	(+)	1	7
Neurological involvement																		
Muscle weakness	(+)	(+)	(−)	(+)	(+)	(+)	(+)	(+)	(−)	(+)	(−)	(+)	(−)	(+)	(−)	(+)	3	8
Numbness	(−)	(+)	(−)	(−)	(−)	(+)	(−)	(+)	(−)	(−)	(−)	(+)	(−)	(+)	(−)	(+)	0	6
Headache	(−)	(+)	(−)	(+)	(+)	(+)	(−)	(+)	(−)	(−)	(+)	(+)	(+)	(+)	(+)	(+)	4	7
Vertigo	(−)	(−)	(−)	(+)	(−)	(+)	(−)	(−)	(−)	(−)	(−)	(+)	(−)	(+)	(+)	(+)	1	5
Dizziness	(−)	(+)	(−)	(+)	(+)	(+)	(−)	(+)	(−)	(−)	(+)	(+)	(+)	(+)	(+)	(+)	4	7
Seizure	(−)	(−)	(−)	(−)	(−)	(+)	(−)	(−)	(−)	(−)	(−)	(−)	(−)	(+)	(−)	(+)	0	3
Blurred vision	(−)	(−)	(−)	(−)	(−)	(−)	(−)	(−)	(−)	(−)	(−)	(+)	(+)	(+)	(−)	(+)	1	3
Urinary retention	(−)	(−)	(−)	(−)	(−)	(−)	(−)	(−)	(−)	(−)	(−)	(−)	(−)	(−)	(−)	(+)	0	1
Others																		
Dyspnea	(+)	(+)	(−)	(−)	(+)	(+)	(−)	(+)	(−)	(+)	(−)	(+)	(−)	(+)	(−)	(+)	2	7
Chest pain	(+)	(+)	(−)	(−)	(+)	(+)	(−)	(+)	(−)	(+)	(−)	(+)	(−)	(+)	(−)	(+)	2	7
Abdominal pain	(+)	(+)	(−)	(+)	(+)	(+)	(−)	(−)	(−)	(+)	(+)	(+)	(−)	(+)	(−)	(+)	3	7
Hematochezia	(−)	(−)	(−)	(−)	(−)	(−)	(−)	(−)	(−)	(+)	(−)	(+)	(−)	(−)	(−)	(+)	0	3
Menorrhea	(−)	(−)	(−)	(−)	(−)	(−)	(−)	(−)	(−)	(−)	(−)	(−)	(−)	(−)	(−)	(+)	0	1
Bone pain	(−)	(−)	(−)	(−)	(−)	(−)	(−)	(−)	(−)	(−)	(−)	(−)	(−)	(−)	(+)	(+)	1	1
Insomnia	(−)	(+)	(−)	(+)	(−)	(−)	(−)	(−)	(−)	(−)	(−)	(+)	(−)	(+)	(−)	(+)	0	5

On laboratory examination, cryofibrinogen was positive and cryoglobulin was negative in seven children (87.5%). Three patients with α1 anti-trypsin checked had negative results (Table 3). In addition, anti-thrombin III was increased in all patients. The other frequent laboratory abnormality was abnormal protein S (87.5%), increased protein C (5/8, 62.5%), decreased C3 (4/8; 50%), and abnormal C4 (4/8; 50%). Other immunologic results had relatively lower proportion of abnormal values. None of the eight children had hepatitis B or C virus infection (Table 2).

**Table 2:** Immunological laboratory examinations

Case	1	2	3	4	5	6	7	8	No. Positive/No. All checked	(%)
Cryofibrinogen	(+)	(+)	(+)	(+)	(+)	(+)	(+)	(+)	8/8	(100)
Cryoglobulin	(+)	(−)	(−)	(−)	(−)	(−)	(−)	(−)	0/8	(0)
α1-antitrypsin	ND	ND	(−)	ND	ND	(−)	ND	(−)	0/3	(0)
PT/aPTT(initial)	(−)	(−)	(−)	(−)	(−)	(−)	(−)	(−)	0/8	(0)
Bleeding time	(−)	ND	ND	ND	ND	ND	ND	ND	0/1	(0)
IgA	(−)	(−)	(−)	(−)	(−)	(−)	ND	(−)	0/7	(0)
Platelet count	(−)	(−)	(−)	(−)	(−)	(−)	(−)	(−)	0/7	(0)
vWF	ND	(−)	I	(−)	(−)	I	(−)	(−)	2/7	(28.5)
C3	(−)	D	(−)	(−)	D	(−)	D	D	4/8	(50)
C4	(−)	(−)	(−)	D	(−)	D	I	D	4/8	(50)
Protein C	I	(−)	(−)	I	I	(−)	I	I	5/8	(62.5)
Protein S	I	(−)	D	D	D	D	D	I	7/8	(87.5)
Anti-thrombin III	I	I	I	I	I	I	I	I	8/8	(100)
Lupus anticoagulant	(−)	ND	(+)	(−)	(+)	(−)	(−)	(−)	2/7	(28.5)
RA factor	(−)	(−)	(+)	(−)	(−)	(+)	(−)	(+)	3/8	(37.5)
Antinuclear Factor	(−)	(−)	(−)	(−)	(−)	(−)	(−)	(+)	1/8	(12.5)
Anti-cardiolipin IgG	(−)	(−)	(−)	(−)	(+)	(−)	(−)	(−)	1/8	(12.5)
Anti-cardiolipin IgM	(−)	(−)	(−)	(−)	(+)	(−)	(−)	(−)	1/8	(12.5)
anti-ENA (all)	(−)	(−)	(−)	(−)	(+)	(−)	(−)	(−)	1/8	(12.5)
Anti CCP	ND	ND	ND	(−)	(−)	(−)	(−)	(−)	0/5	(0)
ANCA-C	(−)	(−)	ND	ND	(−)	(−)	(−)	(−)	0/6	(0)
ANCA-P	(−)	(−)	ND	ND	(−)	(−)	(−)	(−)	0/6	(0)
Anti-β2 GP1 IgG	(−)	(−)	(−)	(−)	(−)	(−)	(−)	(−)	0/8	(0)
Anti-β2 GP1 IgM	(−)	(−)	(−)	(−)	(−)	(−)	(−)	(−)	0/8	(0)
Anti-ds DNA	(−)	(−)	(−)	(−)	(−)	(−)	(−)	(−)	0/8	(0)
Anti-SSA	(−)	(−)	(−)	(−)	(−)	(−)	(−)	(−)	0/8	(0)
Anti-SSB	(−)	(−)	(−)	(−)	(−)	(−)	(−)	(−)	0/8	(0)

*CCP* cyclic citrullinated peptide, *ANCA-C* cytoplasmic anti-neutrophil cytoplasmic antibody, *ANCA-P* peri-nuclear anti-neutrophil cytoplasmic antibody, *Anti-β2 GP1* Anti-β2-glycoprotein I, *ENA* extractable nucleic antigen, *SSA* Sjögren’s-syndrome-related antigen-A, *Anti-SSB* Sjögren’s-syndrome-related antigen-B

**Fig. 2:**
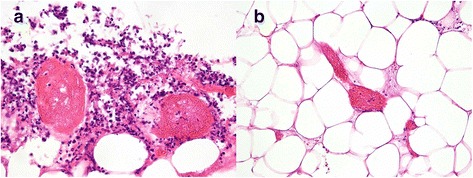
Skin histopathology showed micro-thrombi and deposition within the lumen of blood vessel (**a**, **b**) (Case 7) (400X, H&E stain)

**Fig. 3:**
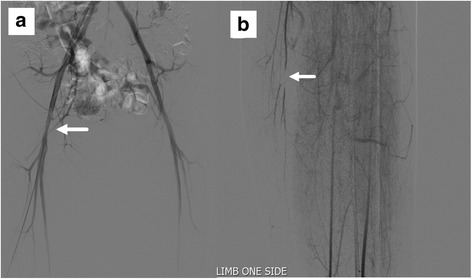
Angiogram demonstrated narrowing on the right femoral artery (black arrow) (Case 7; **a**) and incomplete obstruction of medium-sized arteries on the knee (white arrow) (Case 4; **b**)

Four patients received skin biopsy and one revealed micro-thrombi on small arterioles (Fig. 2). Angiograms were performed on four patients and all revealed abnormal findings that included vessel narrowing or decreased arterial flow rate on the lower extremities, renal arteries, or celiac trunks (Table 3, Fig. 3).

**Table 3:** Systemic involvement and underline disease

Case No.	1	2	3	4	5	6	7	8	No. Abnormal/Total	%
Skin biopsy	(+)	(−)	ND	ND	(+)	ND	ND	(−)	2/4	50
Renal involvement									3/8	37.5
Proteinuria	(−)	(−)	(−)	(−)	(+)	(+)	(−)	(−)	2/8	25
Hematuria	(−)	(−)	(−)	(+)	(+)	(+)	(−)	(−)	3/8	37.5
Renal insufficiency	(−)	(−)	(−)	(−)	(−)	(−)	(−)	(+)	1/8	12.5
Arthritis	(+)	(+)	(+)	(+)	(+)	(+)	(+)	(+)	8/8	100
Thrombotic events	(−)	(−)	(+)	(+)	(−)	(+)	(+)	(+)	5/8	62.5
Angiogram	ND	ND	(+)	(+)	ND	(+)	ND	(+)	4/4	100
Respiratory involvement										
Pulmonary embolism	(+)	ND	(−)	ND	(−)	ND	(−)	ND	1/4	25
ARDS	(+)	(−)	(+)	(+)	(−)	(−)	(+)	(−)	4/8	50
Neurological involvement										
Multineuritis	(+)	(−)	(+)	ND	(−)	(−)	(−)	(+)	3/7	42.9
Brain MRI	(−)	(−)	(+)	(+)	ND	(−)	(+)	(+)	4/7	57.1
Spine MRI	ND	ND	(−)	ND	ND	ND	ND	(−)	0/2	0
SSEP	ND	(−)	(−)	ND	ND	(−)	ND	ND	0/3	0
EMG	ND	(−)	ND	ND	ND	ND	(+)	ND	1/2	50
EEG	ND	(+)	(−)	ND	ND	ND	(+)	(+)	3/4	75
BAEP	ND	(−)	ND	ND	ND	(−)	(−)	(+)	1/4	25
VEP	ND	ND	ND	ND	ND	ND	(+)	(+)	2/2	100
Associated disease										
Autoimmune disease	CG	(−)	JIA	JIA	JIA, JDM, SLE	JIA	B, SLE, JIA	CM, JIA, B	6/8	75
HCV/HBV infection	(−)	(−)	(−)	(−)	(−)	(−)	(−)	(−)	0	0

(+) = abnormal; (−) = normal; ND = not done

*ARDS* acute respiratory distress syndrome, *NCV* nerve conduction velocity, *EMG* electromyogram, *SSEP* somatosensory evoked potential, *BAEP* brainstem auditory evoked potential, *VEP* visual evoked potential, *HCV* hepatitis C virus infection, *HBV* hepatitis B virus infection, *CG* cryoglobulinemia, *JIA* juvenile idiopathic arthritis, *JDM* juvenile dermatomyositis, *SLE* systemic lupus erythematosus, *B* Behcet’s disease, *CM* Cutaneous mastocytosis

All eight patients had neurologic symptoms, the most common of which was muscle weakness (Table 1). On electro-physiologic examinations, NCV revealed neuropathic change on three of seven (42.9%) patients, compatible with multi-neuritis. The VEP showed prolonged P100 in two patients who complained of blurred vision or diplopia. The EEG showed spikes or cortical dysfunction on three of four patients (75%) who presented with chronic headache or consciousness disturbance. Three patients who received SSEP examinations had normal findings (Table 2).

**Fig. 4:**
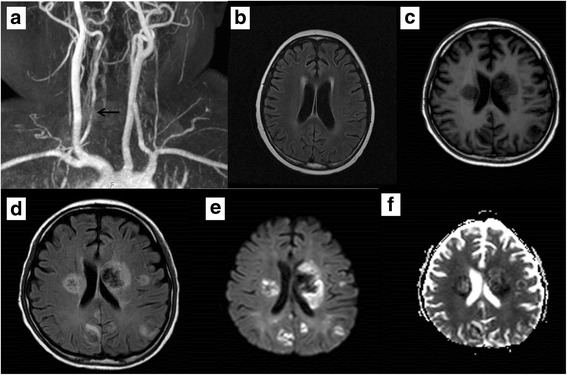
Brain magnetic resonance imaging (MRI). **a**) MR angiogram showed right vertebral artery narrowing (arrow) (Case 3). **b**) Axial T2-weighted flair MRI imaging demonstrated hyper-intense lesion on the peri-ventricular white matter (TR/TE: 9002/135.4 ms) (Case 3). **c**–**f**) Acute hemorrhagic encephaomyelitis (Case 8). Axial T2-weighted flair showed multiple hyper-intense with central hypo-intense lesions on the thalamus and white matter (T1-weighted flair: TR/TE: 2000/20 ms; T2-weighted flair: TR/TE: 8000/125 ms; ADC and DWI: TR/TE: 6000/72.89 ms)

On imaging studies, four of seven (57.1%) patients who underwent brain MRI had abnormal findings (Fig. 4). One patient revealed vertebral artery narrowing. Two others had increased T2-weighted signal intensity on the thalamus or white matter. Another revealed multiple hyper-intense with central hypo-intense lesions on T2-paired images on the thalamus and white matter, indicating hemorrhage in the demyelinating lesions. Diffusion-weighted imaging revealed restriction (increasing signal) in this area (Fig. 4).

Other frequent involvements were the gastrointestinal (GI) and respiratory systems. Although seven of the eight patients had complained of recurrent abdominal pain, several kinds of GI examinations (e.g. plain abdominal X-ray, abdominal sonogram, and upper or lower GI series) were usually normal. One patient underwent abdominal MRI, which revealed inflammatory change in the middle jejunal loop, probably related to vasculitis.

In the respiratory system, seven patients complained of dyspnea or chest pain. One presented with pulmonary emboli on lung perfusion scan. Four patients suffered from acute respiratory distress syndrome and expired in the end stage. Three (37.5%) had renal involvement, with proteinuria, hematuria, or renal insufficiency.

As regards associated disease, One patient (12.5%) was primary cryofibrinogenemia (Cases 2) and six had co-morbid JIA, SLE, Behcet’s disease, JDM, and cutaneous mastocystosis (Table 3). Three of these six had mixed autoimmune disease. There were five (62.5%) mortalities (Table 1).

## Discussion

Cryofibrinogenemia is very rare in children. In review of literature, only Geest et al. reported primary cryofibrinogenemia in a family with 2 children. They presented with only mild skin manifestations as purpura and bullae [9]. Compared to common clinical manifestations, purpura and arthralgia are more prevalent than in previous review articles (purpura 0–78%; arthralgia 6.25–58%). Nevertheless, muscle weakness has not been previously mentioned [2]. This kind of disease is difficult to diagnose in children because initial complaints are variable and non-specific. We approached this disease from the perspective of purpura. The platelet count of all patients was normal. According to the follow-up non-thrombocytopenia purpura survey, all patients exhibited normal PT/aPTT. Thus, we considered vWF disease, platelet dysfunction, and vascular disease to have caused purpura. Although PLT dysfunction and vWF diseases are typically congenital, those cases diagnosed in the patients were acquired. Therefore, the purpura caused by vascular diseases was examined. Although Henoch–Schonlein purpura (HSP) is the most common disease causing vascular purpura in children, we confirmed diagnoses of cryofibrinogenemia after an extensive survey [10]. Other children complained of long-term and recurring pain at multiple sites on the body, with symptoms such as arthralgia, myalgia, bone pain, numbness, abdominal pain, and headache; however, these have even been regarded as psychiatric problems.

In laboratory studies, an immunologic role for cryofibrinogenemia is suggested by the possible presence of immunoglobulins in the precipitate and of complement deposits in vessel walls. There are excess circulating immunoglobulins or immune complexes produced during pathologic conditions (e.g. infection, cancer, inflammatory, and collagen diseases) [2]. Although most cases (87.5%) in this study is secondary cryofibrinogenemia, the abnormal rate of autoimmune antibody is relatively low. In our study, autoantibodies are usually negative in the initial stage and became positive after treatment. Thus, we speculated that autoantibodies are fixed in the vessel walls and released into circulation after treatment; however, this requires further study.

What mechanisms cause the increased protein C and anti-thrombin III levels in cryofibrinogenemia? This issue has not been discussed in previous studies. We speculated that it may reflect compensation for a temporary hypercoagulatory state of the body. Furthermore, there is no case of cryofibrinogenemia with HBV or HCV infection in our study. This may be due to the HBV vaccination prophylaxis and low possibility of viral exposure in children in Taiwan.

In terms of neurologic involvement, the proportion of multi-neuritis in this study is 42.9%, slightly higher than in previous reports (4.1–22%) [2]. The patients here have higher proportions of CNS involvement, which is rare in previous studies. Only one previous study mentions central nerve system involvement in cryofibrinogenemia. Dunsker et al. reported primary cryofibrinogenemia in a patient who presented with pseudo-tumor cerebri. He suffered from headache, blurred vision, and papillary edema. Venograms revealed cerebral venous thrombosis [11].

In this study, patients with CNS involvement presented as large artery narrowing, parenchymal involvement, and hemorrhage in demyelinating lesions on the white matter and basal ganglion. The last is compatible with acute hemorrhagic encephalomyelitis (AHEM), considered a hyper-acute sub-form of acute disseminated encephalomyelitis. It is frequently a fulminant inflammatory hemorrhagic demylination of the CNS white matter. Death from brain edema is common within one week of the onset of encephalopathy. The pathogenesis is known as acute vasculitis with subsequent vessel occlusion [12]. The patient here (Case 8) with AHEM presented with consciousness disturbance and repeated seizures. Herpes simplex virus infection is identified by laboratory examinations. Thus, these three kinds of brain MRI findings are all compatible with vasculopathy.

In respiratory system involvement, seven of eight (87.5%) patients have complained of dyspnea and chest pain. But chest x-rays often reveal no abnormality. Pulmonary embolism can occur (Case 1), but ARDS is the most frequent complication causing mortality. On the other hand, abdominal pain is also a frequent complaint but determining the underlying problem is difficult. Abdominal vasculitis is proven by angiography or MRI. The rate of renal involvement in this study is also slightly higher than in previous reports (4–22%) [2].

## Conclusions

In conclusion, this study reports on the presentations of cryofibrinogenemia, which is rare in children. Most pediatric cases are associated with autoimmune diseases and have more severe and complex presentations. Although rarely reported previously, CNS involvement is common in our study. When children complain of persistent unexplained pain on multiple sites with purpura or ecchymosis, cryofibrinogenemia should be considered and further investigated.

## Abbreviations

aPTT: activated thromboplastin time
ARDS: acute respiratory distress syndrome
BAEP: brainstem auditory evoked potential
CNS: central nervous system
EMG: electromyogram
GI: gastrointestinal
HBsAg: Hepatitis B virus surface antigen
HBV: hepatitis B virus
HCV: hepatitis C virus
IgA: immunoglobulin A
JDM: juvenile dermatomyositis
JIA: juvenile idiopathic arthritis
MRI: magnetic resonance imaging
NCV: nerve conduction velocity
PT: prothrombin Time
SLE: systemic lupus erythematosus
SSEP: somato-sensory evoked potential
VEP: visual evoked potential
vWF: von Willebrand factor
